# Association Between TLR9-Mediated Interferon-Alpha Production and Short-Term Risk of Common Cold: A Pilot Observational Study

**DOI:** 10.3390/tropicalmed11100274

**Published:** 2026-09-30

**Authors:** Yukiko Kato, Truong Tuyet Mai, Nghiem Nguyet Thu, Osamu Kanauchi, Yusuke Ushida

**Affiliations:** 1 Institute of Health Sciences, Kirin Holdings Co., Ltd., Fujisawa 251-8555, Japan; 2 National Institute of Nutrition, Hanoi 12638, Vietnam

**Keywords:** plasmacytoid dendritic cells, type I interferon, common cold, upper respiratory tract infections

## Abstract

Upper respiratory tract infections (URTIs) are highly prevalent and impose substantial individual and societal burdens. Plasmacytoid dendritic cells (pDCs) are major producers of type I interferons (IFNs) with antiviral activity. This study investigated whether CpG oligodeoxynucleotide-induced IFN-α production by peripheral blood mononuclear cells (PBMCs), a marker of pDC activity, was associated with subsequent occurrence of the common cold. Data from 41 healthy adults in the placebo arm of a randomized controlled trial were analyzed. CpG-induced IFN-α production was measured before a 4-week observation period, and participants were dichotomized into high and low IFN-α production groups based on the median value. The occurrence of common cold events and cold symptoms was assessed using daily self-reported symptom scores and body temperature records. Seven participants developed the common cold, all in the low group. The crude risk difference between the low and high groups was 0.333 (Miettinen–Nurminen 95% CI, 0.148–0.546; Fisher’s exact *p* = 0.009). The low group also showed higher risks for fever, plugged nose, and sore throat, while findings for the other symptoms were less conclusive. As a hypothesis-generating pilot study, these findings suggest that low TLR9-mediated IFN-α production may be associated with increased susceptibility to URTIs in healthy adults.

## 1. Introduction

Upper respiratory tract infections (URTIs), including the common cold, influenza, acute pharyngitis, and acute sinusitis, are among the prevalent contagious diseases worldwide [1,2]. Although URTIs such as the common cold are typically self-limiting, they impose a substantial socioeconomic burden through reduced quality of life and productivity losses due to absenteeism [2,3]. Moreover, pandemic respiratory infections such as the 1918 influenza and COVID-19 have shown high mortality rates and significant effects on life expectancy [4,5,6], highlighting the need for effective preventive measures. Vaccination and other public health interventions are crucial for controlling these viral infections. Additionally, priority should be given to populations at a high risk of exposure (e.g., healthcare workers) or severe disease (e.g., older adults and individuals with comorbidities) [7,8]. However, individual susceptibility to viral infections remains overlooked, which can be attributed to the lack of validated methods for predicting individual-level resistance to viral pathogens and risk of URTIs.

Plasmacytoid dendritic cells (pDCs) are antigen-presenting cells that are crucially involved in linking innate and adaptive immunity. They are the principal producers of type I interferons (IFNs) and contribute to early host defense against viral and bacterial infections [9]. Viral RNA or DNA is detected by toll-like receptors (TLRs), including TLR7 and TLR9, which trigger robust production of type I IFNs [10,11,12,13,14,15,16,17]. Type I IFNs induce the expression of antiviral effector molecules that inhibit viral replication and promote degradation of viral nucleic acids [13,14,17]. Moreover, type I IFNs promote the differentiation and maturation of myeloid dendritic cells, activate CD8+ T cells, and enhance Th1 and natural killer cell responses, which eventually shape early adaptive and humoral immunity [17]. In addition to their role in host defense against infection, pDCs have also been implicated in vaccine-induced immune responses through type I IFN-mediated mechanisms [18].

Accordingly, there has been extensive research on the involvement of pDCs and type I IFNs in respiratory viral infections. Severe influenza and COVID-19 are associated with reduced pDC numbers, impaired IFN-α production, and decreased expression of activation markers on dendritic cells, with some alterations persisting for months after SARS-CoV-2 infection [15,16,19,20,21,22,23]. Furthermore, decreased type I IFN responses have been correlated with severe dengue disease during the acute phase [24,25]. Prospective and experimental studies also support a protective role of pDCs in host defense against respiratory infections. For example, higher circulating pDC levels during infancy have been associated with a lower subsequent risk of respiratory tract infections, wheezing, and asthma development, suggesting that intrinsic differences in pDC frequency may precede disease onset rather than result from infection in childhood [26]. In addition, an experimental study has demonstrated that pDCs contribute to antiviral immune responses and protection against viral infection [27].

However, the role of pDCs in viral infections is complex. Although pDCs are major producers of type I IFNs, and numerous studies support their antiviral functions, accumulating evidence suggests that their contribution may vary according to the pathogen, tissue, and disease stage [28]. In some settings, pDC-derived IFNs may be partially compensated for by other cellular sources, whereas in other contexts, they may even contribute to immunopathology [28]. Therefore, the overall significance of pDC function in determining susceptibility to naturally occurring viral respiratory infections remains incompletely understood [16,28].

Despite increasing evidence regarding the role of pDCs in viral infections, important uncertainties remain regarding whether baseline pDC function is associated with subsequent occurrence of naturally acquired respiratory infections. Given the limited prospective evidence available in healthy adults, a descriptive and hypothesis-generating pilot observational study was conducted to explore potential associations between TLR9-mediated IFN-α production, a functional surrogate marker of pDC activity, and subsequent common cold outcomes.

## 2. Materials and Methods

### 2.1. Study Design and Participants

**Figure 1:**
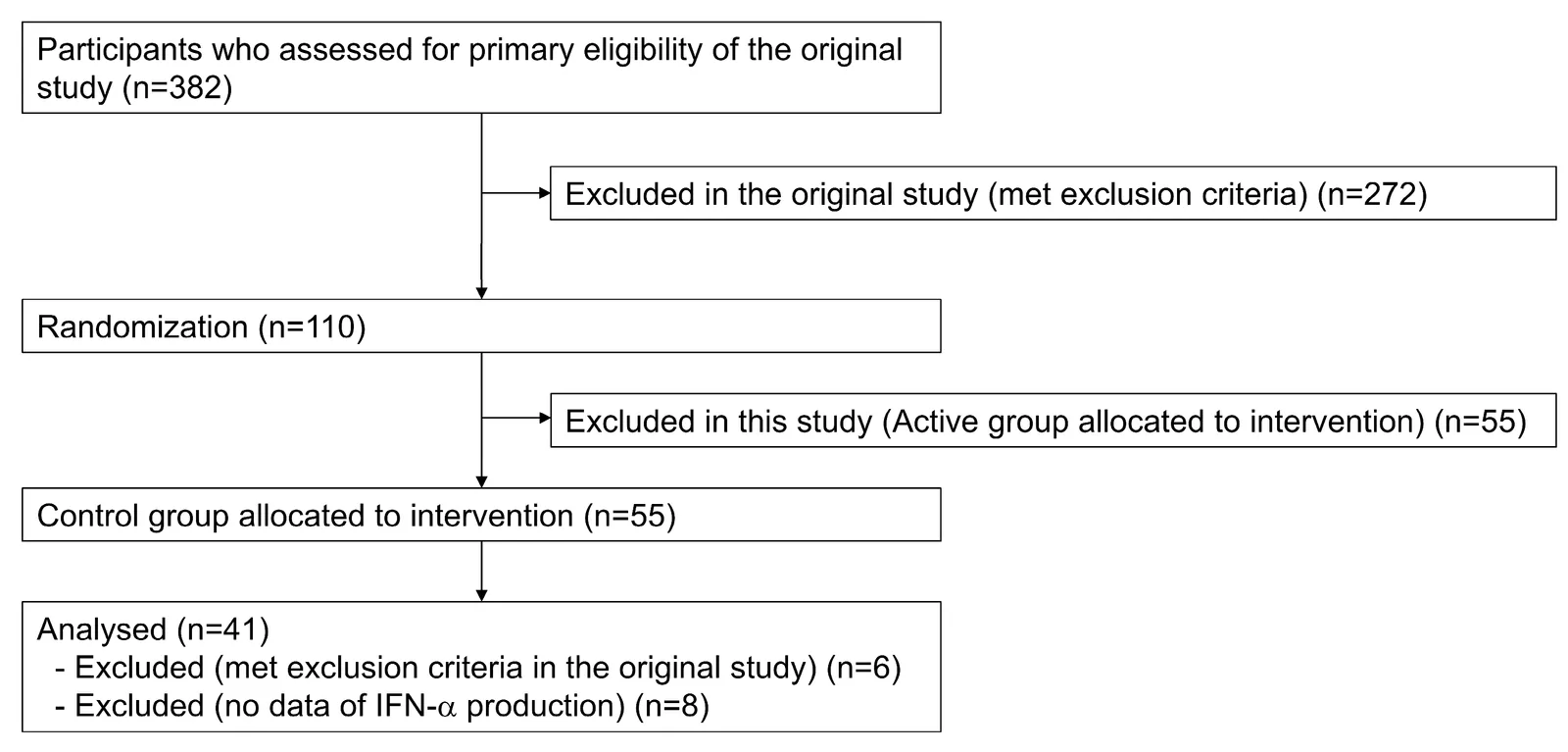
Flow diagram of the study selection process.

This study was a prospective, exploratory, secondary analysis of data derived from the placebo arm of a randomized controlled trial performed in healthy Vietnamese medical students [29]. The parent study was registered with the University Hospital Medical Information Network Clinical Trial Registry (Registration No. UMIN000053690). The parent study was conducted at Hai Phong University of Medicine and Pharmacy (Hai Phong, Vietnam) from November 2023 through December 2024. Participants were recruited between 30 November and 1 December 2023, and written informed consent was obtained from all study participants. It initially enrolled 110 students; among them, 55 were randomized to the placebo group. After excluding participants with missing baseline IFN-α production data or those who did not meet eligibility criteria, 41 participants (Figure 1) were included. Participants with underlying acute and/or chronic sickness from respiratory disease and/or gastrointestinal disease were not eligible for enrollment. Individuals regularly using immunosuppressive drugs or with known allergies to milk and/or soy (contained within the study products) were also excluded. Information on the history of other chronic infectious diseases or allergic diseases was not collected in the parent study. Participants were required to be free from active SARS-CoV-2 infection at enrollment, and at least 4 weeks had elapsed since recovery in those with a previous history of COVID-19. Information on COVID-19 infection occurring more than four weeks before enrollment was not systematically collected in the parent study. Detailed descriptions of the parent study protocol are available in the original report [29]. Data collected in the parent study were accessed for this secondary analysis between 15 July and 2 December 2025. All data were anonymized prior to analysis, and the authors had no access to identifiable participant information during or after data collection.

### 2.2. Measurement of IFN-α Production as an Exposure

Peripheral blood mononuclear cells (PBMCs) were collected at baseline before the observation period; further, IFN-α production from PBMCs had been measured in the parent study as previously described [29]. Briefly, PBMCs (2 × 10^5^ cells/mL) were stimulated with 0.05 μM CpG oligodeoxynucleotide (CpG ODN) 2216 (InvivoGen, San Diego, CA, USA), followed by harvesting of culture supernatants after 24 h. IFN-α protein concentrations in PBMC supernatants were quantified using a human IFN-α ELISA kit (E-EL-H6125, Elabscience, Wuhan, China) following the manufacturer’s instructions. Upon thawing, PBMC viability consistently exceeded 80% [29]. According to the manufacturer, the assay is designed to detect all subtypes of human IFN-α. The lower limit of quantification of the assay was 4.69 pg/mL. Values below the lower limit of quantification were retained as reported by the assay and included in all analyses without imputation or substitution.

### 2.3. Measurement of Common Cold Incidence as Outcome Variables

Participants self-recorded their daily health status using a four-point scoring system based on the modified Wisconsin Upper Respiratory Symptom Survey-21 (WURSS-21) and by measuring body temperature for four weeks (Table S1) [29,30]. Based on the parent study, a body temperature ≥37.5 °C was classified as fever [29]. Using a previously described modified definition, a common cold event was prospectively defined in the study protocol as a day in which the sum score of seven symptoms from the modified WURSS-21 was ≥4 and fever was present; an individual symptom was considered present on days when its score was ≥2 [31]. The primary outcome was the occurrence of the common cold during the observation period. Secondary outcomes included the occurrence of each cold symptom. Body temperature data were missing for three participants for 1–3 days; accordingly, days with missing temperature measurements were classified as no fever. There were no missing data regarding the modified WURSS-21 score.

### 2.4. Identification of Cell Types Producing IFN-α

PBMCs from three donors, which were obtained from Research Donors Ltd. (London, UK), were stimulated with CpG for 20 h, treated with Brefeldin A Solution (BioLegend, San Diego, CA, USA) for 6 h, harvested, and analyzed as per the study protocol [32]. Briefly, the Zombie Red Fixable Viability Kit (BioLegend) was utilized to define live cells, with cells incubated with Fc block (BD Biosciences, San Jose, CA, USA). Extracellular surface staining was performed using the antibodies listed in Table S2; furthermore, pDCs and mDCs were defined as lineage−CD11c−HLA-DR+CD123+ and lineage−CD11c+ HLA-DR+ CD123−, respectively. Cellular fixation and permeabilization were performed using the Cytofix/Cytoperm Fixation/Permeabilization kit (BD Biosciences) following the manufacturer’s instructions; additionally, intracellular staining for IFN-α was performed. Cells were analyzed using the Spectral Cell Analyzer ID7000 (SONY, Tokyo, Japan), followed by analysis using FlowJo software (version 10.6.0, BD Biosciences). Figure S1a shows the gating strategy, which is consistent with a previous study [33].

### 2.5. Statistical Analysis

Participants were dichotomized into the high and low IFN-α groups based on the median baseline IFN-α concentration (10.1 pg/mL) in the CpG-stimulated PBMC supernatants.

To examine the relationship between IFN-α production and the risk of the common cold, between-group comparisons of the incidence of the common cold and cold symptoms during a 4-week observation period were performed. Because no common cold cases occurred in the high group, risk differences (RDs) were selected as the primary effect measure, and Fisher’s exact test was additionally used as an exact inferential approach for the primary comparison. Crude RDs were calculated from the observed proportions. For the primary crude comparison, Fisher’s exact test was used to obtain the inferential *p*-value, and Miettinen–Nurminen score-based confidence intervals were calculated for the RD. Sex-adjusted RDs and 95% confidence intervals (CIs) were estimated using the modified least squares regression approach proposed by Cheung [34]; these adjusted analyses were considered sensitivity analyses. Specifically, a Gaussian generalized linear model with an identity link (linear probability model) was fitted, and robust standard errors were used to estimate confidence intervals and *p*-values. As a sensitivity analysis for the primary outcome, participants were additionally categorized into tertiles according to baseline IFN-α production, and the association was re-evaluated across tertiles. A two-sided score-based trend test within a conditional inference framework was performed to evaluate the trend in common cold occurrence across increasing tertiles of baseline IFN-α production, with ordered scores of 1, 2, and 3 assigned to the LOW, MIDDLE, and HIGH tertiles, respectively. In addition, Firth penalized logistic regression was performed to assess the robustness of the findings under sparse data conditions and potential quasi-complete separation [35]. The time to the first onset of a common cold event was analyzed using Kaplan–Meier survival curves. Between-group differences were assessed using the stratified log-rank test, with sex specified as the stratification factor. To descriptively evaluate symptom occurrence during the observation period, the cumulative number of days with common cold and each cold symptom was categorized as 0, 1, 2, or ≥3 days and summarized according to baseline IFN-α production group. No formal statistical testing was performed for these exploratory analyses. All statistical analyses were performed using R software (version 4.2.2), and figures were generated using R software (version 4.2.2) or GraphPad Prism (version 10.6.0, GraphPad Software, Boston, MA, USA). This study was reported in accordance with the STROBE statement, and the completed checklist is provided as Supplementary Material [36].

## 3. Results

**Figure 2:**
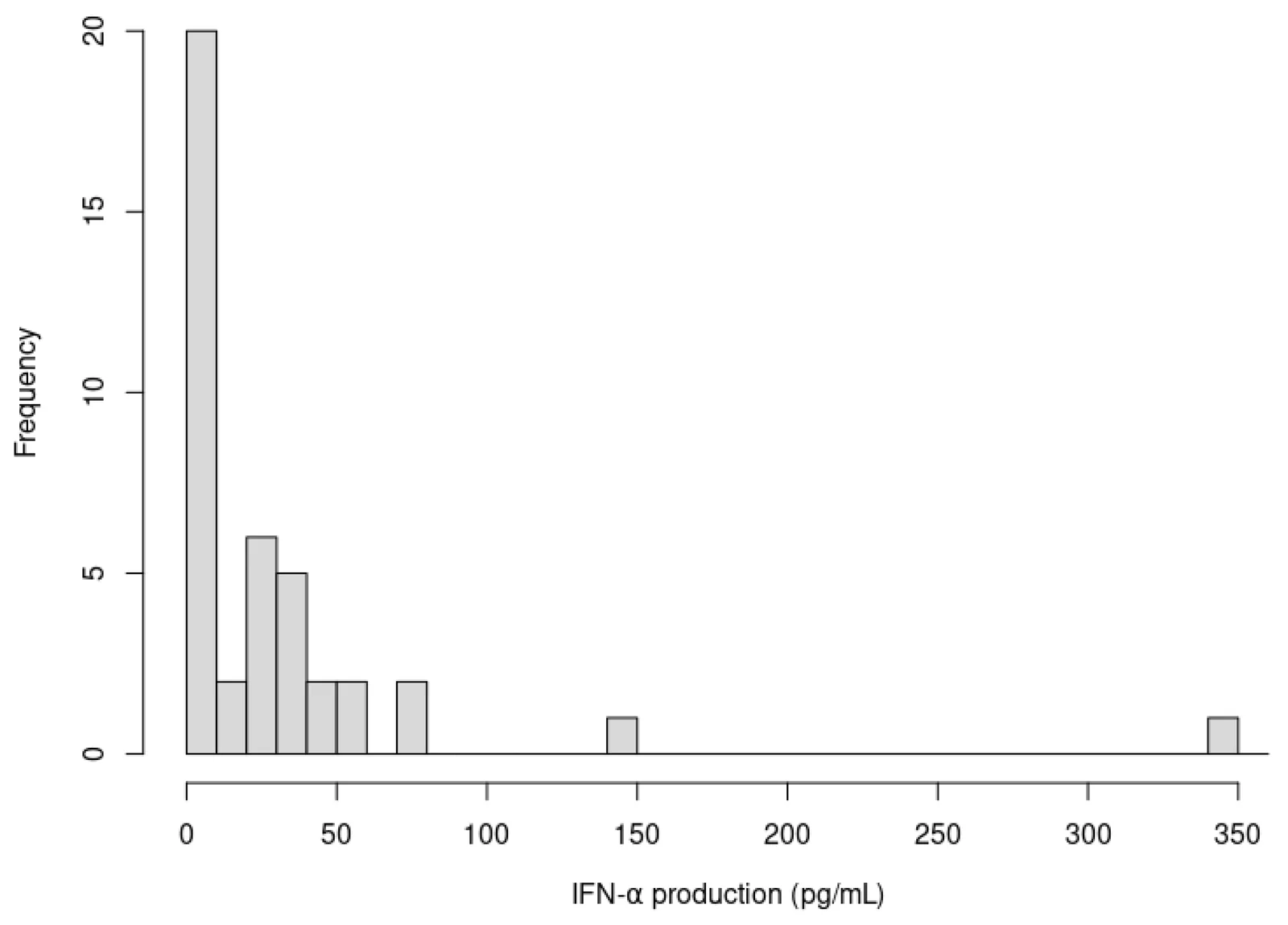
Distribution of CpG-induced IFN-α production. Histogram of IFN-α concentrations (pg/mL) in culture supernatants of PBMCs stimulated with CpG for 24 h. The histogram was constructed using 10 pg/mL wide bins, and each bar indicates the number of participants whose IFN-α concentration fell within the corresponding interval. The median is 10.1 pg/mL.

Participants were dichotomized into the high and low IFN-α groups based on the median value (10.1 pg/mL) (Figure 2). Eighteen of the 41 participants had baseline IFN-α concentrations below the assay lower limit of quantification (LLOQ; 4.69 pg/mL). Consistent with its use as a functional surrogate of pDC activity, intracellular staining of CpG-stimulated PBMCs showed that IFN-α production was predominantly derived from the pDC compartment, with an average of 97.2% (standard deviation [SD] = 2.9%) of IFN-α-positive cells identified as pDCs (Figure S1b). Table 1 summarizes the baseline characteristics. As shown in Table 1, the sex distribution was balanced in the high group, whereas the low group comprised approximately three times as many men as women. The distribution of IFN-α production was strongly right-skewed, as shown in Figure 2.

During the 4-week observation, seven participants experienced a common cold event. All seven cases occurred in the low group, whereas no cases occurred in the high group. Consistent with this observation, Fisher’s exact test yielded *p* = 0.009, and the risk of the common cold was greater in the low group than in the high group (crude RD = 0.333; 95% CI, 0.148–0.546) (Table 2). In the sex-adjusted sensitivity analysis, the adjusted RD was 0.314 (95% CI, 0.114–0.513; *p* = 0.002) (Table 2), and the sex-adjusted linear probability model generated some fitted probabilities below 0 (minimum, −0.038), whereas none exceeded 1.

In a sensitivity analysis based on tertiles of baseline IFN-α production, the proportions of participants with a common cold were 28.6% (4/14), 21.4% (3/14), and 0.0% (0/13) in the low, middle, and high tertiles, respectively (two-sided score-based trend test, *p* = 0.053) (Table S3). In a further sensitivity analysis using Firth penalized logistic regression, the low group showed higher odds of common cold occurrence than the high group in both the crude analysis [crude odds ratio (OR) = 21.2; 95% CI, 2.27–2830; *p* = 0.004] and the sex-adjusted analysis (adjusted OR = 17.1; 95% CI, 1.82–2280; *p* = 0.009) (Table S4).

Further, low baseline IFN-α production was associated with an increased risk of typical cold symptoms, including fever (crude RD = 0.381; 95% CI, 0.193–0.591; *p* = 0.003; adjusted RD = 0.354; 95% CI, 0.150–0.558; *p* = 0.001), plugged nose (adjusted RD = 0.289; 95% CI, 0.007–0.571; *p* = 0.045), and sore throat (adjusted RD = 0.284; 95% CI, 0.028–0.539; *p* = 0.030) (Table 3). Sneezing occurred in only two participants (one in each group), precluding meaningful comparison between groups. Positive adjusted RDs were also observed for runny nose, cough, and head congestion, but the corresponding confidence intervals were wide. The estimated RDs for sneezing and feeling tired were close to zero.

Additionally, the Kaplan–Meier curves showed earlier common cold events in the low group, whereas no common cold events were observed in the high group during the observation period (stratified log-rank *p* = 0.013) (Figure S2). The descriptive distribution of the cumulative number of days with common cold or cold symptoms also showed a greater proportion of participants with ≥3 days of common cold and several symptoms, particularly fever, runny nose, plugged nose, sore throat, and cough, in the low-IFN-α group than in the high-IFN-α group (Table S5).

## 4. Discussion

Our findings suggest a potential association between low TLR9-mediated IFN-α production and a greater occurrence of common cold and cold-related outcomes during short-term follow-up (Table 2 and Table 3). Although there may be a relationship between URTIs and pDC/type I IFN biology, it remains unclear whether impaired pDC function precedes or results from infection. Our prospective study design allowed an initial evaluation of a potential temporal relationship between TLR9-mediated IFN-α production and the incidence of the common cold. By assessing IFN-α production prior to infection under natural exposure conditions, this study provides hypotheses regarding a possible relationship between innate immune functional status and subsequent common cold occurrence.

Overall, low IFN-α production was associated with an increased RD as well as a greater cumulative number of days of common cold and fever (Table 2, Table 3 and Table S5). However, fever should be interpreted as a nested outcome because it was incorporated into the predefined common cold definition used in the present study. A similar directional pattern was observed in the sensitivity analysis based on tertile categorization (Table S3). However, this pattern should be interpreted cautiously, as the difference in common cold incidence between the LOW and MIDDLE tertiles was modest (28.6% vs. 21.4%), and the apparent trend was largely driven by the absence of common cold events in the HIGH tertile. Firth penalized logistic regression also yielded estimates in the same direction as the primary analysis (Table S4). At the individual symptom level, however, the associations with IFN-α production differed across symptoms. There were observed differences in the RDs for plugged nose and sore throat, but not for runny nose, sneezing, cough, head congestion, or feeling tired (Table 3). For runny nose, cough, and head congestion, the estimates suggested greater risks in the low group, but the confidence intervals were wide and compatible with a broad range of associations. Overall, sneezing occurred in only two participants. The negligible RD for feeling tired may reflect substantial contributions from non-infectious causes [37,38,39]. The descriptive distribution of cumulative symptom days also suggested a greater proportion of participants with ≥3 days of several symptoms, particularly runny nose, plugged nose, sore throat, and cough, among participants with lower IFN-α production (Table S5). However, these observations should be interpreted cautiously because of the limited sample size and the small number of outcome events. In this context, the sex difference in IFN-α production observed in this study is consistent with previous reports describing higher type I IFN responses in women than in men [40,41,42,43]. The factors underlying the inter-individual variability in IFN-α production observed in this study remain unclear. In addition to sex-related differences, previous studies have suggested that genetic background, lifestyle factors, underlying health conditions, and other environmental exposures may influence innate immune function [22,44,45,46,47,48]. Future studies collecting such information may provide further insight into the determinants of variation in TLR9-mediated IFN-α production.

Although the common cold is generally self-limiting, susceptibility to respiratory viral infections varies substantially among individuals. Our findings raise the possibility that functional differences in innate immune responses may contribute to this variability.

This study has certain limitations. First, an important statistical limitation was the very small number of common cold events (*n* = 7). Because the findings were based on only seven outcome events for the primary outcome, even a change in the classification of a single participant could materially affect the estimated associations. For example, reclassifying one event from the LOW group to the HIGH group would change Fisher’s exact *p*-value from 0.009 to 0.093, illustrating the statistical fragility of the observed association. The relatively small number of events may also partly reflect the stringent outcome definition used in the parent study, which required both a symptom score ≥4 and fever. Although this definition was intended to improve the specificity of common cold identification, it may have reduced the number of events captured during follow-up. Therefore, the results should be regarded as descriptive and hypothesis-generating rather than definitive evidence of an association, and confirmation in substantially larger prospective studies is required. Although sensitivity analyses using tertile-based categorization and Firth penalized logistic regression yielded estimates in the same direction as the primary analysis, the confidence intervals remained wide, reflecting the limited precision associated with the small number of events. Furthermore, odds-ratio estimates derived from sparse outcome data should be interpreted cautiously, and the Firth-based estimates should be considered supportive sensitivity analyses rather than confirmatory evidence.

Second, the observation period was limited to four weeks, which may not entirely identify longer-term patterns of the incidence of the common cold. Third, the common cold was assessed based on self-reported symptoms and body temperature records rather than physician diagnosis or virological confirmation. Consequently, the causative pathogens could not be identified, and pathogen-specific analyses could not be performed. Because different respiratory pathogens can vary substantially in their immunopathogenesis, the observed association cannot be attributed to any specific pathogen. Future studies incorporating molecular pathogen diagnostics may provide a more detailed understanding of the relationship between TLR9-mediated IFN-α production and susceptibility to respiratory infections. Another limitation relates to participant exclusions. Eight participants were excluded because baseline IFN-α measurements were unavailable, and six additional participants were excluded because they met the exclusion criteria for the present secondary analysis. Because detailed information on excluded participants was not available from the parent study dataset, formal comparisons between analyzed and excluded participants could not be performed. Although sex as a confounder was adjusted for, our study design impeded collection of information regarding other potential confounders, including background characteristics and living environment. In addition, the proportion of men was higher in the low IFN-α group than in the high IFN-α group (76% vs. 50%). Previous studies have reported higher type I interferon responses in women [40,41,42,43]. Residual confounding related to sex therefore cannot be excluded, and the small number of outcome events may have limited the ability of statistical adjustment to fully account for this imbalance. Therefore, the possibility that factors other than IFN-α production were associated with the incidence of the common cold cannot be excluded. Additionally, the study population comprised Vietnamese medical students with frequent exposure to patients with URTI in clinical settings, which may limit the generalizability of the findings in terms of geographic region, age group, and exposure profile. Another limitation is related to the exposure measure (IFN-α production induced by CpG ODN stimulation). CpG ODN activates innate immune responses via TLR9 and primarily reflects DNA virus-associated signaling, whereas many common URTI pathogens are RNA viruses. Therefore, it remains unclear whether CpG-induced IFN-α production fully reflects IFN responses to the wide range of viruses that cause URTI. IFN-α production was assessed using circulating PBMCs obtained from peripheral blood. Therefore, the measured response may not fully reflect pDC activity within lymphoid tissues or at mucosal sites where respiratory viral infections are initiated. Finally, 18 of 41 participants had IFN-α concentrations below the assay lower limit of quantification (LLOQ; 4.69 pg/mL), and the entire interquartile range of the LOW group (0.7–3.1 pg/mL) was below the LLOQ. Values below the LLOQ were retained as reported by the assay and included in all analyses without imputation or substitution. Accordingly, IFN-α production could not be meaningfully quantified as a continuous variable throughout the lower portion of its distribution, and the observed exposure contrast may partly reflect a comparison between participants with undetectable and detectable IFN-α production rather than solely low and high concentrations.

Despite these constraints, this study has notable strengths. Daily self-recording of cold symptoms and body temperature had minimal missing data, which likely minimized information bias. Furthermore, given that individual cold symptoms were recorded and analyzed separately in conjunction with an overall common cold definition, we could explore associations between IFN-α production and individual cold symptoms. The use of standardized self-reported symptom recording further facilitates comparison with and replication in other clinical studies employing similar outcome measures. Notably, since the exposure was assessed prior to infection and the observation was prospective under natural exposure conditions, the analysis provides an opportunity to explore potential relationships between baseline immune function and subsequent common cold outcomes.

## 5. Conclusions

In conclusion, this descriptive and hypothesis-generating pilot observational study suggests that low TLR9-mediated IFN-α production may be associated with increased susceptibility to URTIs. Given the small number of outcome events and the resulting uncertainty, these preliminary findings require confirmation in larger prospective studies.

## Figures and Tables

**Table 1:** Characteristics of participants in the high- and low-IFN-response groups.

Characteristic	HIGH (*n* = 20)	LOW (*n* = 21)
Age, mean (SD), years	21.9 (0.8)	21.6 (0.5)
Sex, no. (%)		
Male	10 (50)	16 (76)
Female	10 (50)	5 (24)
IFN-α production, median (IQR), pg/mL	33.8 (28.3–56.3)	2.6 (0.7–3.1)

IFN, interferon; HIGH, high-IFN-response group; LOW, low-IFN-response group; IQR, interquartile range. The category boundary (median of IFN-α production) of the groups was 10.1 pg/mL.

**Table 2:** Risk difference in the incidence of the common cold.

Group	*n*	Event	Crude RD (95% CI, *p*-Value)	Adjusted RD (95% CI, *p*-Value)
LOW	21	7	0.333 (0.148 to 0.546, 0.009)	0.314 (0.114 to 0.513, 0.002)
HIGH	20	0	Reference	Reference

RD, risk difference; CI, confidence interval; HIGH, high-IFN-response group; LOW, low-IFN-response group. Crude 95% confidence intervals were calculated using the Miettinen–Nurminen score method without the small-sample variance correction. Fisher’s exact test was used for inference in the primary crude comparison. Adjusted analyses were performed using modified least squares regression with adjustment for sex.

**Table 3:** Risk difference in the incidence of each common cold symptom.

Cold Symptoms	Group	*n*	Event	RD (95% CI, *p*-Value)
Fever	LOW	21	8	Crude RD = 0.381 (0.193 to 0.591, 0.003) Adjusted RD = 0.354 (0.150 to 0.558, 0.001)
	HIGH	20	0	Reference
Runny nose	LOW	21	9	Adjusted RD = 0.224 (−0.061 to 0.509, 0.123)
	HIGH	20	4	Reference
Plugged nose	LOW	21	9	Adjusted RD = 0.289 (0.007 to 0.571, 0.045)
	HIGH	20	3	Reference
Sneezing	LOW	21	1	Adjusted RD = -0.0243 (−0.168 to 0.119, 0.739)
	HIGH	20	1	Reference
Sore throat	LOW	21	8	Adjusted RD = 0.284 (0.028 to 0.539, 0.030)
	HIGH	20	2	Reference
Cough	LOW	21	7	Adjusted RD = 0.214 (−0.056 to 0.483, 0.121)
	HIGH	20	2	Reference
Head congestion	LOW	21	6	Adjusted RD = 0.203 (−0.015 to 0.420, 0.068)
	HIGH	20	2	Reference
Feeling tired	LOW	21	7	Adjusted RD = 0.0135 (−0.276 to 0.303, 0.927)
	HIGH	20	6	Reference

RD, risk difference; CI, confidence interval; HIGH, high-IFN-response group; LOW, low-IFN-response group. For fever, crude RD is presented because no fever events occurred in the HIGH group. The 95% CI was calculated using the Miettinen–Nurminen score method without the small-sample variance correction, and Fisher’s exact test was used for inference. The adjusted estimate is presented as a sensitivity analysis. Fever is a component of the primary common cold case definition and therefore represents a nested rather than an independent outcome. For all other symptoms, adjusted risk differences were estimated using modified least squares regression with adjustment for sex.

## Data Availability

The de-identified participant-level dataset analyzed in the present study is not publicly available because the data are subject to confidentiality and data-sharing restrictions under the parent study protocol and the collaboration agreements among the participating institutions. Data generated in the parent study are treated as confidential information under the collaboration agreements and therefore cannot be disclosed to third parties without the necessary approval of the participating institutions. As stated in the Data Availability Statement of the parent study, requests for access to the data should be directed to the corresponding author of the parent study.

## Supplementary Materials

The following supporting information can be downloaded at https://www.mdpi.com/article/10.3390/tropicalmed11100274/s1, Figure S1: Identification of IFN-α-producing cells following CpG stimulation of total PBMCs; Figure S2: Kaplan–Meier curves for time to common cold onset; Table S1: Upper respiratory tract infection score in the modified WURSS-21; Table S2: Antibody list used for flow cytometry to identify the cell types of IFN-α-producing cells; Table S3: Common cold occurrence according to tertiles of baseline IFN-α production; Table S4: Firth penalized logistic regression analysis of common cold occurrence; Table S5: Distribution of the cumulative number of days of common cold and cold symptoms according to baseline IFN-α production group.
