# Supplementary material for: Association Between TLR9-Mediated Interferon-Alpha Production and Short-Term Risk of Common Cold: A Pilot Observational Study
Source: Trop Med Infect Dis. 2026 Sep 30;11(10):274. doi: 10.3390/tropicalmed11100274 (PMC13709414; doi:10.3390/tropicalmed11100274)
Supplement: Supplementary file 1 [file tropicalmed-11-00274-s001.zip › tropicalmed-4469547-supplementary.pdf]

Supplementary Material

Supplementary Figures

(a)

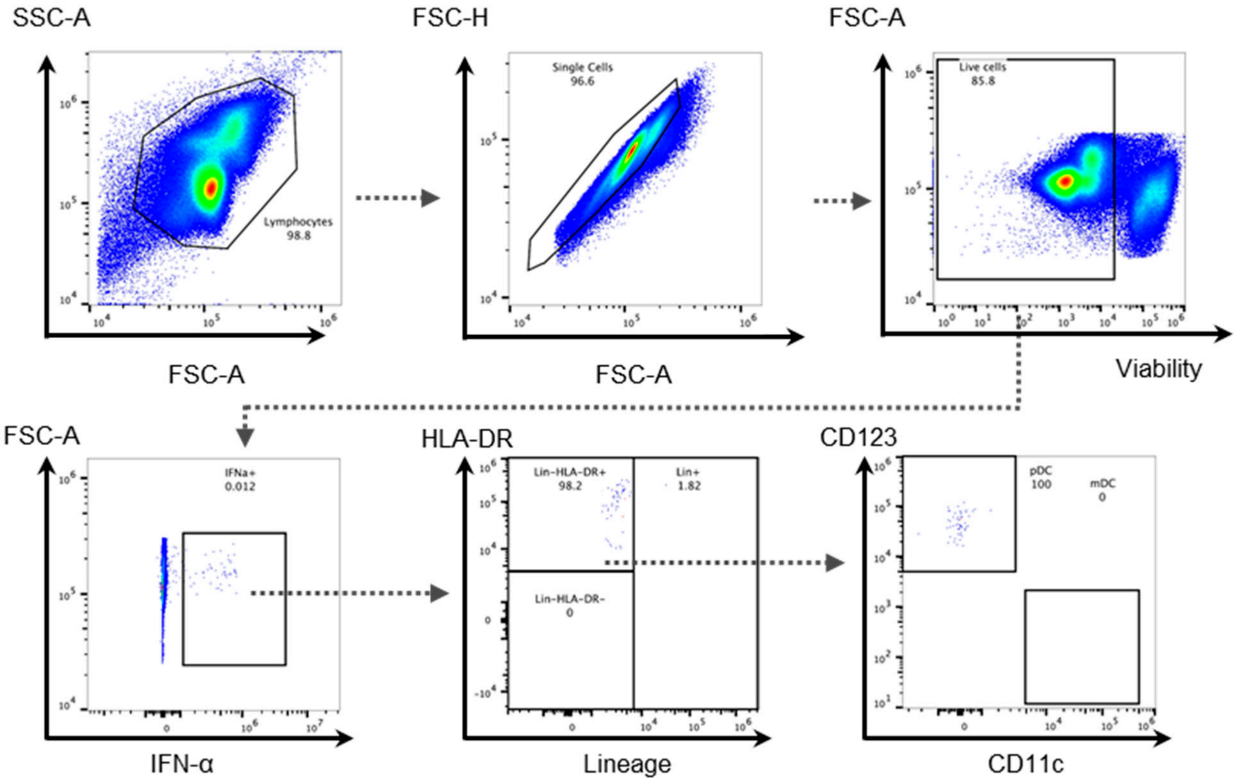

(b)

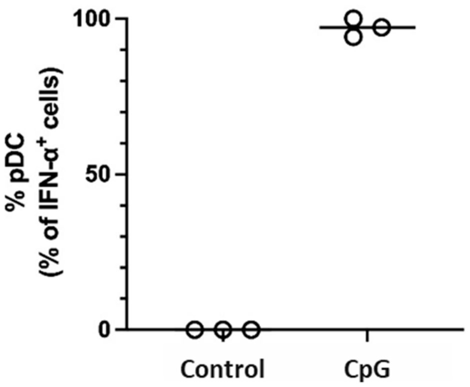

Figure S1. Identification of IFN-α-producing cells following CpG stimulation of total PBMCs

(a) Gating strategy for IFN-α-producing cells. PBMCs were gated on lymphocytes, singlets, live cells, and intracellular IFN-α+ cells (IFN-α producing cells). IFN-α+ cells were identified by gating lineage, HLA-DR, CD123, and CD11c. pDCs were identified as lineage−CD11c−HLA-DR+CD123+. (b) The percentage of pDCs among IFN-α^+ cells was quantified under unstimulated and stimulated conditions based on the gating strategy shown in Figure S1. IFN-α^+ cells were not detected in the control group. CpG-induced IFN-α^+ cells were predominantly identified within the pDC compartment, with a mean of 97.2% (standard deviation, 2.9%). PBMCs from three independent donors were analyzed, with each symbol representing an individual donor.

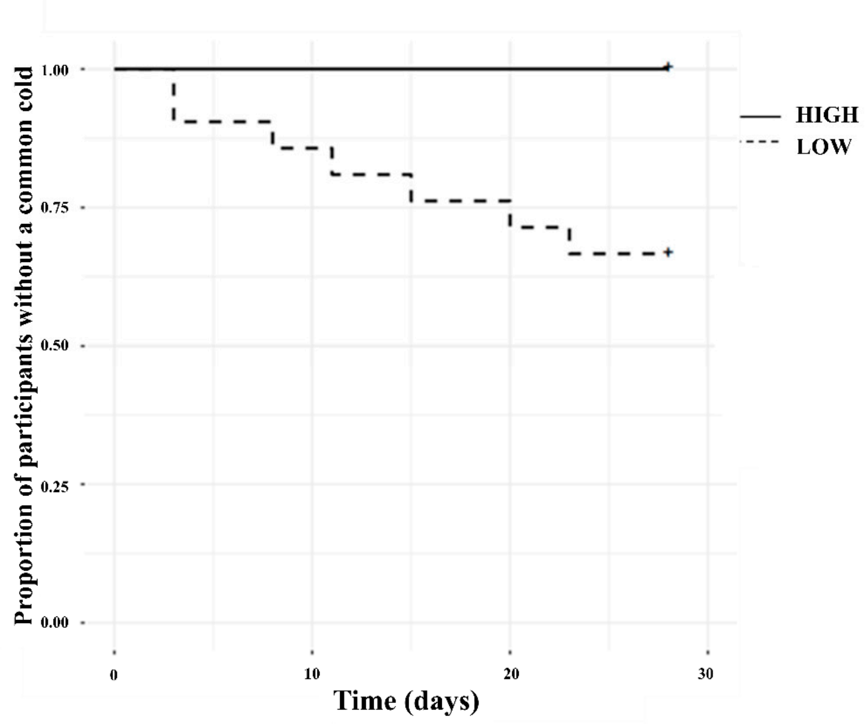

Figure S2. Kaplan–Meier curves for time to common cold onset

Kaplan–Meier curves show the proportion of participants who remained free from a common cold over the 4-week observation period. Participants were stratified into the HIGH and LOW groups based on IFN-α production at baseline. Between-group differences were assessed using a log-rank test stratified by sex (P = 0.013).

HIGH, high IFN response group; LOW, low IFN response group

Supplementary Tables

Table S1. Upper respiratory tract infections score in the modified WURSS-21

	Score	0	1	2	3
**URID score (modified WURSS-21)**	**Runny nose**	None	Slight	Moderate	Severe
	**Plugged nose**	None	Slight	Moderate	Severe
	**Sneezing**	None	Slight	Moderate	Severe
	**Sore throat**	None	Slight	Moderate	Severe
	**Cough**	None	Slight	Moderate	Severe
	**Head congestion**	None	Slight	Moderate	Severe
	**Feeling tired**	None	Slight	Moderate	Severe

WURSS-21, Wisconsin Upper Respiratory Symptom Survey-21

Table S2. Antibody list used for flow cytometry to identify the cell types of IFN-α producing cells

**Antibody name**	**Manufacturer**	**Catalogue No**	**Extra/Intra**
FITC anti-human Lineage Cocktail (CD3, CD14, CD19, CD20, CD56)	BioLegend	348701	Extracellular
Alexa Fluor 700 anti-human CD11c	BioLegend	337220	Extracellular
Brilliant Violet 570 anti-human HLA-DR	BioLegend	307638	Extracellular
Brilliant Violet 510 anti-human CD123 Antibody	BioLegend	305412	Extracellular
PE Mouse anti-Human IFN-α[2b]	BD Bioscience	560097	Intracellular

Table S3. Common cold occurrence according to tertiles of baseline IFN-α production

Tertile	n	Event	Proportion (%)
LOW	14	4	28.6
MIDDLE	14	3	21.4
HIGH	13	0	0.0

HIGH, highest tertile of baseline IFN-α production; MIDDLE, middle tertile of baseline IFN-α production; LOW, lowest tertile of baseline IFN-α production.

Table S4. Firth penalized logistic regression analysis of common cold occurrence

Group	n	Event	Crude OR (95% CI, P-value)	Adjusted OR (95% CI, P-value)
LOW	21	7	21.2 (2.27 to 2830, 0.004)	17.1 (1.82 to 2280, 0.009)
HIGH	20	0	Reference	Reference

OR, odds ratio; CI, confidence interval; HIGH, high IFN response group; LOW, low IFN response group

Odds ratios were estimated using Firth penalized logistic regression to account for sparse outcome data and potential quasi-complete separation. Adjusted estimates were obtained with adjustment for sex.

Table S5. Distribution of the cumulative number of days of common cold and cold symptoms according to baseline IFN-α production group

Cold symptoms	Group	n	0 days	1 day	2 days	≥ 3 days
Common cold	LOW	21	14	3	2	2
	HIGH	20	20	0	0	0
Fever	LOW	21	13	2	3	3
	HIGH	20	20	0	0	0
Runny nose	LOW	21	12	3	1	5
	HIGH	20	16	1	1	2
Plugged nose	LOW	21	12	4	2	3
	HIGH	20	17	0	1	2
Sneezing	LOW	21	20	1	0	0
	HIGH	20	19	0	0	1
Sore throat	LOW	21	13	6	0	2
	HIGH	20	18	1	1	0
Cough	LOW	21	14	1	2	4
	HIGH	20	18	1	1	0
Head congestion	LOW	21	15	5	0	1
	HIGH	20	18	0	1	1
Feeling tired	LOW	21	14	3	2	2
	HIGH	20	14	2	1	3

HIGH, high IFN response group; LOW, low IFN response group
